# Case Report: Sticky bone-assisted autogenous tooth transplantation for compromised alveolar recipient sites: a case series

**DOI:** 10.3389/fbioe.2026.1926597

**Published:** 2026-08-25

**Authors:** Shenghui Zhou, Lin Chen, Yuyao Huang, Zi’ao Dong

**Affiliations:** 1 Department of General and Emergency Dentistry, Stomatological Hospital of Southern Medical University, Guangzhou, Guangdong, China; 2 Guangzhou Liwan District Stomatology Hospital, Guangzhou, Guangdong, China

**Keywords:** alveolar defect management, autogenous tooth transplantation, maxillary sinus-floor elevation, periodontal ligament, sticky bone

## Abstract

**Background:**

Autogenous tooth transplantation is one of the treatment options for replacing missing or non-restorable teeth in appropriately selected patients, depending on patient-related factors, donor-tooth availability, and recipient-site conditions. Sticky bone was prepared by combining particulate deproteinized bovine bone mineral with autologous fibrin glue and was applied to the recipient-site osseous defects at the time of transplantation. We report a case series of autogenous tooth transplantation using sticky bone–assisted recipient-site management in four complex scenarios.

**Case presentation:**

Four patients underwent autogenous tooth transplantation with sticky bone grafting. Indications included vertical ridge defects, irregular extraction sockets, and a maxillary sinus-floor defect with an intact Schneiderian membrane. All procedures followed standard surgical protocols. Ethical approval was obtained and all patients provided written informed consent.

**Results:**

At the final follow-up examinations, ranging from 12 to 30 months, all four transplanted teeth remained *in situ* and clinically functional. Radiographic root-surface changes of varying degrees were observed in all four cases; however, qualitative comparison of the available serial images showed no clear progression of inflammatory or replacement root resorption, ankylosis-related changes, or progressive periapical pathology during the observation periods.

**Conclusion:**

In this descriptive case series, sticky bone was used as an adjunct for managing peri-root defects during autogenous tooth transplantation at four anatomically compromised recipient sites. All transplanted teeth remained clinically functional during follow-up periods ranging from 12 to 30 months. Although radiographic root-surface changes were observed, no clear progression of inflammatory or replacement resorption was identified during the available follow-up. However, because of the small sample size, case heterogeneity, non-standardized radiographic assessment, and absence of a control group, the observed outcomes cannot be attributed to sticky bone.

## Introduction

Autogenous tooth transplantation (ATT) is a treatment option for replacing missing or non-restorable teeth because it preserves the periodontal ligament (PDL) and supports alveolar bone maintenance through physiological stimulation (Park et al., 2010). It may be particularly useful in growing patients, for whom implant placement is generally contraindicated during continued alveolar growth. With appropriate case selection and surgical technique, ATT can provide functional and esthetic rehabilitation (Nimcenko et al., 2013).

Successful ATT depends on preservation of the PDL and adequate recipient-site support (Chugh et al., 2012; Suwanapong et al., 2021). Insufficient alveolar volume or buccal plate deficiency may compromise initial stability and periodontal healing (Tan et al., 2023). Although guided bone regeneration is commonly used for alveolar reconstruction, membrane exposure, limited structural stability, and inadequate space maintenance may reduce its predictability in irregular defects (Elgali et al., 2017). Sticky bone, prepared by combining particulate graft material with autologous blood or platelet concentrates, may improve graft handling, cohesion, and dimensional stability (Aghaloo et al., 2025). These considerations provide a rationale for investigating sticky bone-assisted recipient-site management in ATT, although direct clinical evidence remains limited.

Based on the current literature, ATT remains a biologically favorable treatment option, but published studies continue to emphasize recipient-site conditions and other prognostic factors rather than adjunctive biomaterials specifically designed for transplantation sites (Kafourou et al., 2017). Sticky bone has been described in regenerative dentistry as a moldable graft complex composed of particulate bone material and autologous platelet concentrate or fibrin-based adhesive, with reported advantages in graft cohesion, handling, and stability (Xie et al., 2024; Yang et al., 2025). However, direct clinical evidence for sticky bone-assisted recipient site management in ATT appears limited, and the currently available literature on ATT does not yet establish this approach as a standard adjunctive protocol. Therefore, the present study aimed to describe the clinical application and preliminary outcomes of sticky bone-assisted recipient site management in autogenous tooth transplantation through a retrospective technical case series.

## Materials and methods

### Study design, setting, and case selection

This study was designed as a retrospective, purposively selected technical case series. Four patients who had undergone autogenous tooth transplantation with adjunctive sticky bone grafting were purposively selected to illustrate the application of this technique in different compromised recipient-site conditions. All patients were treated at the Department of General and Emergency Dentistry, Stomatological Hospital of Southern Medical University, Guangzhou, Guangdong Province, China. The patients’ clinical records, intraoral photographs, surgical documentation, and available radiographic examinations were retrospectively reviewed.

The cases were not consecutively enrolled. They were selected on the basis of the availability of sufficiently complete clinical, surgical, and radiographic documentation and the presence of distinct anatomical or reconstructive challenges requiring recipient-site management. The selected cases represented different clinical scenarios, including vertical alveolar defects, irregular extraction sockets, a maxillary sinus-floor bony defect, and an impacted anterior tooth associated with alveolar deficiency. The purpose of the case selection was to illustrate the clinical and technical application of sticky bone during autogenous tooth transplantation rather than to estimate treatment success or determine the independent effectiveness of sticky bone. Routine comprehensive hematological testing was not performed.

The cases were heterogeneous with respect to donor-tooth morphology, root configuration, impaction status, recipient-site location, and defect morphology. They were therefore presented individually and were not pooled for comparative or efficacy analysis.

The clinical and procedural characteristics of the four cases, including donor-tooth morphology, recipient-site condition, surgical timing, stabilization method, digital assistance, endodontic management, and follow-up duration, are summarized in Table 1.

**TABLE 1:** Clinical and procedural characteristics of the four autogenous tooth transplantation cases.

Case	Age/Sex	Donor tooth and root status	Recipient site	Recipient-site condition	Surgical timing	Tooth stabilization	3D-printed replica	Endodontic management	Latest follow-up
1	31/F	Tooth 48; fully formed roots; multirooted	Tooth 46 region	Distal and buccal alveolar wall defects following extraction of a non-restorable, infected tooth 46	Delayed transplantation, 4 weeks after extraction of tooth 46	Combined suture and ligature-wire fixation; removed after 7 days	No	Endodontic access at 2 weeks; definitive root canal obturation at 3 months	12 months
2	23/F	Tooth 38; fully formed roots; multirooted	Tooth 36 region	Circumferential vertical osseous defect associated with severe alveolar bone loss around tooth 36	Immediate transplantation after extraction and debridement of tooth 36	Suture fixation alone; removed after 7 days	No	Endodontic access at 2 weeks; definitive root canal obturation at 3 months	12 months
3	23/F	Tooth 18; fully formed roots; multirooted	Tooth 16 region	Loss of the bony sinus floor and palatal alveolar wall; intact but exposed Schneiderian membrane	Delayed transplantation, 2 weeks after extraction of tooth 16	Metal-wire fixation; removed after 7 days	No	Definitive root canal obturation at 3 months; initiation date not consistently documented	12 months
4	18/F	Impacted tooth 21; fully formed, single and markedly dilacerated root	Anatomically planned tooth 21 position in the maxillary anterior arch	Vertical and horizontal alveolar deficiency following unsuccessful surgical-orthodontic traction	Immediate repositioning after surgical extraction of the impacted tooth	Stainless-steel orthodontic ligature-wire fixation; removed after 7 days	Yes	Intraoperative root-end preparation and retrograde filling; orthograde treatment initiated at 7 days with Vitapex; definitive obturation at 5 months	30 months

F, female; 3D, three-dimensional; NR, not recorded; RCT, root canal treatment.

### Surgical protocol

All procedures were performed under local anesthesia. Donor teeth were extracted atraumatically with meticulous preservation of the periodontal ligament whenever possible. Recipient sites were prepared according to the morphology of each defect. Sticky bone was used solely for the management of recipient-site osseous defects and was not relied upon as the primary mechanical stabilizer of the transplanted teeth. Primary stability was achieved through adaptation of the donor tooth to the prepared recipient socket and was supplemented, when required, by case-specific suture, ligature-wire, metal-wire, or orthodontic-wire fixation. Wound closure was performed using a 3-0 braided silk non-absorbable suture, 75 cm in length, mounted on a 25-mm round-bodied needle (ETHICON W570; Ethicon, Johnson & Johnson Medical Devices). The same suture material and needle specification were used in all four cases.

### Preparation of the AFG, CGF, and sticky bone

Immediately before grafting, a total of 10 mL of peripheral venous blood was collected into separate blood-collection tubesfor the preparation of autologous fibrin glue (AFG 5 mL surface-untreated tube) and concentrated growth factor (CGF 5 mL surface-treated tube) (Jiangsu Kangjian, China). The tubes were balanced and centrifuged in a low-speed benchtop centrifuge (Pulaimeidun, China) using a variable-speed program of 2,400–2,700 rpm for 12 min without interruption. After centrifugation, the whole upper liquid autologous fibrin glue (AFG) fraction was aspirated and mixed with 500 mg of particulate deproteinized bovine bone mineral (Geistlich Bio-Oss®, small granules; Geistlich Pharma AG, Switzerland). The volume of recovered AFG was not measured in the original clinical records; therefore, an exact graft-to-AFG ratio could not be determined. The concentrated growth factor (CGF) clot was subsequently separated from the lower red blood cell layer. The remaining CGF clot was compressed to form an autologous fibrin membrane for use when clinically indicated. The graft components were allowed to polymerize for approximately 5 min, producing a cohesive and moldable sticky bone graft. The prepared graft was trimmed according to the dimensions of the recipient-site defect and applied immediately. Most of the prepared graft was gently adapted to the defect as clinically required, without forceful packing.

### Donor-tooth morphology and crown management

Donor-tooth morphology and recipient-site dimensions were assessed clinically and radiographically before transplantation. The evaluation included crown dimensions, root number and configuration, and the available mesiodistal and buccolingual space at the recipient site. Donor teeth with favorable dimensional and morphological compatibility were selected, with ipsilateral teeth from the same arch preferred when clinically available. When necessary, conservative enamel recontouring was performed to improve proximal adaptation or eliminate occlusal interference. All transplanted teeth were maintained free of premature functional contact during the initial healing period. Definitive restorative modification with an onlay or full-coverage crown was considered only when a clinically significant crown-form discrepancy remained after periodontal healing.

### Postoperative management

Postoperative antibiotics were prescribed on a case-specific basis. Cases 2–4 received amoxicillin (0.5 g, three times) for 3 days (Segura-Egea et al., 2018). In Case 1, cefuroxime axetil (0.25 g, twice daily) for 3 days was prescribed because the patient had previously experienced marked gastrointestinal intolerance to amoxicillin; this was not a documented penicillin allergy. A soft diet, avoidance of chewing on the transplanted tooth, and careful oral hygiene were recommended during the initial healing period. Fixation was removed 7 days after surgery in all cases after satisfactory clinical stability had been confirmed. Premature occlusal contacts were eliminated when necessary. In Case 3, the patient was instructed to avoid nose blowing and to sneeze with the mouth open, and other activities that could increase sinus pressure for 1 month. No acute postoperative infection, wound dehiscence, graft exposure, or tooth loss was recorded.

### Outcome assessment and follow-up

Tooth survival was defined as the transplanted tooth remaining *in situ* and clinically functional at the latest follow-up. Treatment success was defined as tooth survival without symptoms or infection, with physiological mobility, no abnormal metallic percussion sound, healthy periodontal soft tissues, and no radiographic evidence of progressive inflammatory root resorption, progressive replacement resorption or ankylosis-related changes, or progressive periapical pathology. Treatment failure was defined as loss or extraction of the transplanted tooth, inability of the tooth to maintain clinical function, or a decision to extract the tooth because of an unfavorable prognosis (Plotino et al., 2021) (Table 2).

**TABLE 2:** Follow-up findings of the four transplanted teeth.

Case	Follow-up	Clinical findings	Radiographic findings	Status
Case 1: 48→46	12 months	Asymptomatic and functional; physiological mobility; healthy periodontal tissues	Visible PDL space and periradicular bone healing; root-surface changes were present, without definite progressive resorption, ankylosis-related changes, or progressive periapical pathology	Retained and functional
Case 2: 38→36	12 months	Asymptomatic and functional; physiological mobility; healthy periodontal tissues	Stable surrounding alveolar bone and visible PDL space; root-surface changes were present, without definite progressive resorption, ankylosis-related changes, or progressive periapical pathology	Retained and functional
Case 3: 18→16	12 months	Asymptomatic and functional; physiological mobility; healthy periodontal tissues	Maintained sinus-floor contour and visible PDL space; root-surface changes were present, without definite progressive resorption, ankylosis-related changes, or progressive periapical pathology	Retained and functional
Case 4: Repositioned tooth 21	30 months	Asymptomatic and functional; physiological mobility; healthy periodontal tissues	Root-end filling remained visible; root-surface changes were present, without definite progressive resorption, ankylosis-related changes, or progressive periapical pathology	Retained and functional

PDL, periodontal ligament.

### Consent for publication

Written informed consent was obtained from all patients for the treatment procedures and for the publication of their anonymized clinical information, intraoral photographs, and radiographic images.

## Case reports

### Case 1

#### Patient information and chief complaint

A 31-year-old woman presented to our department with a chief complaint of a fractured and severely decayed right mandibular posterior tooth associated with recurrent buccal abscess formation for more than 1 month. She reported that the tooth had fractured during mastication approximately 1 month earlier, followed by intermittent purulent discharge from the buccal gingiva.

#### Medical and dental history, clinical and radiographic examination

The patient’s medical history was noncontributory. She denied any history of hypertension, diabetes mellitus, or drug allergy. Extraoral examination revealed symmetrical facial contours, normal mouth opening and mandibular movement, and no tenderness or clicking of the temporomandibular joints.

**FIGURE 1:**
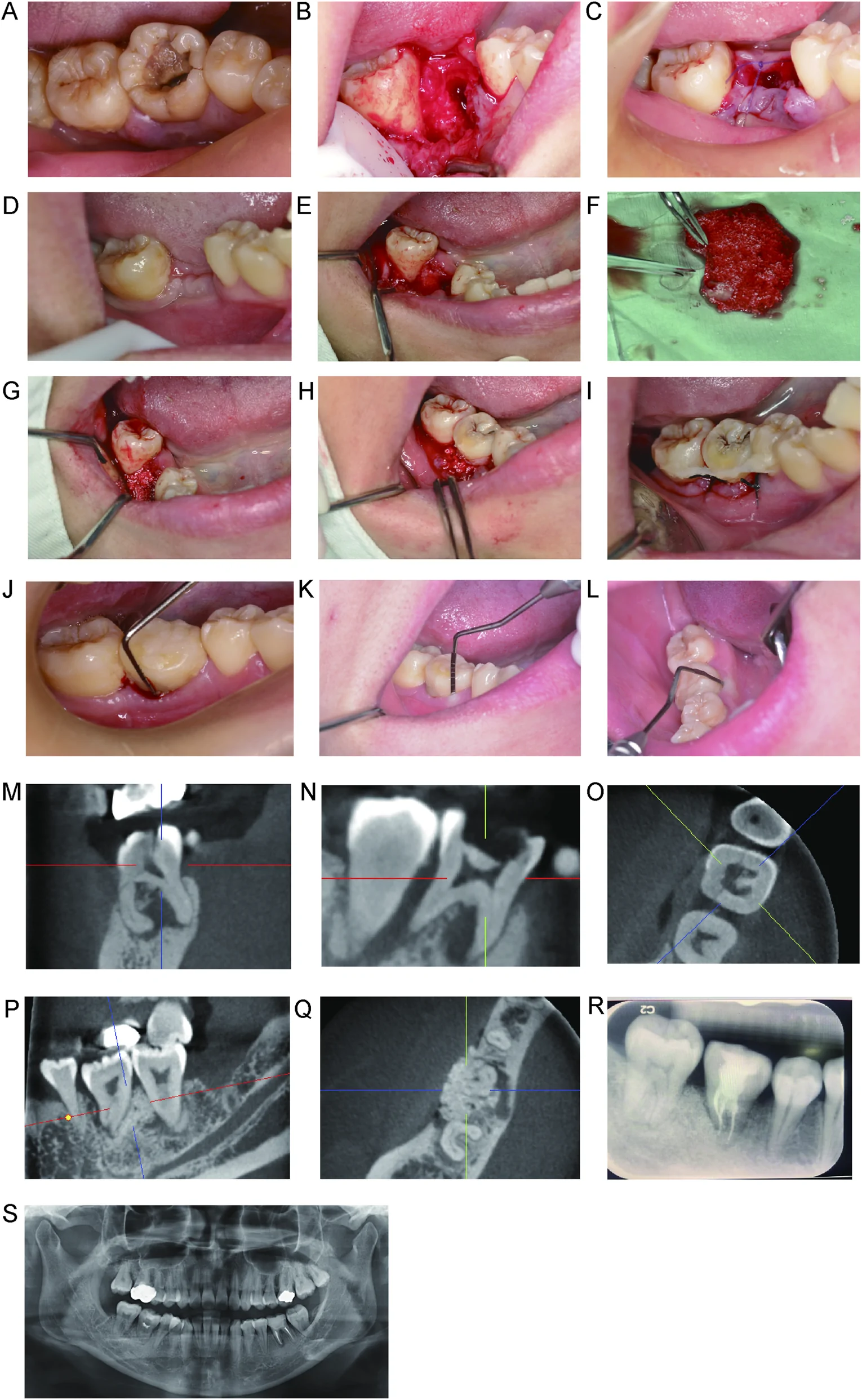
Clinical photographs and radiographic images of Case 1 showing autogenous tooth transplantation of tooth 48 to the tooth 46 recipient site. **(A)** Initial intraoral photograph of tooth 46 showing extensive crown destruction. **(B)** Minimally traumatic extraction of tooth 46. **(C)** Suturing of the extraction socket after removal of tooth 46. **(D)** Gingival condition at the tooth 46 site 4 weeks after extraction. **(E)** Flap elevation and preparation of the recipient socket at the tooth 46 site. **(F)** Preparation of sticky bone. **(G)** Placement of sticky bone into the recipient socket and alveolar defect at the tooth 46 site. **(H)** Minimally traumatic extraction of tooth 48 and insertion into the prepared tooth 46 recipient socket. **(I)** Stabilization of transplanted tooth 48 at the tooth 46 site. **(J)** Periodontal probing and gingival condition 3 months after autogenous tooth transplantation. **(K,L)** Periodontal probing and gingival condition at the 1-year follow-up. **(M–O)** Preoperative CBCT images before autogenous tooth transplantation. **(P,Q)** Immediate postoperative CBCT image after autogenous tooth transplantation. **(R)** Periapical radiograph after completion of root canal treatment 3 months after transplantation. **(S)** Panoramic radiograph at the 1-year follow-up. ATT, autogenous tooth transplantation; CBCT, cone-beam computed tomography.

Intraoral examination showed extensive carious destruction of tooth 46 with pulp chamber exposure and only thin residual tooth walls remaining. A mesiodistal fracture line extended subgingivally along the residual crown. The buccal gingiva adjacent to tooth 46 was erythematous and swollen, with a fistulous tract at the gingival margin from which purulent exudate could be expressed. Tooth 48 was present and exhibited marginal gingival erythema with soft debris accumulation (Figure 1A).

CBCT revealed a periradicular radiolucency around tooth 46 and mesial alveolar bone loss adjacent to tooth 47, extending to the apical third (Figures 1M–O).

#### Diagnosis

Tooth 46: crown-root fracture with chronic apical periodontitis.

Tooth 48: nonfunctional third molar.

#### Treatment options and decision-making

Three treatment options were discussed with the patient: extraction of tooth 46 followed by orthodontic mesialization of tooth 48 to close the edentulous space; extraction of tooth 46 with simultaneous alveolar ridge preservation followed by delayed implant-supported restoration after 3 months; or extraction of tooth 46 followed by autogenous transplantation of tooth 48 into the recipient site.

Considering the prolonged treatment duration required for orthodontic space closure and the higher cost of implant therapy, the patient elected autogenous tooth transplantation. The planned treatment consisted of staged extraction of tooth 46 with socket debridement and primary closure, full-mouth scaling 1 week later, delayed reconstruction of the recipient-site defect with sticky bone and simultaneous transplantation of tooth 48 after 4 weeks, endodontic access 2 weeks after transplantation, definitive root canal treatment after stabilization of tooth mobility, and regular follow-up.

#### Surgical procedure

In the first stage, tooth 46 was atraumatically extracted (Figure 1B). The extraction socket was thoroughly debrided and closed with nonresorbable sutures, and hemostasis was achieved with cotton-roll compression for 30 min (Figure 1C). Full-mouth scaling was performed 1 week later.

At the 4-week follow-up, the buccal fistula had resolved completely (Figure 1D). A full-thickness mucoperiosteal flap was elevated, revealing resorption of the distal bony wall of the tooth 46 socket extending to the apical level of tooth 47, together with a buccal bony wall defect. The recipient socket was prepared with surgical burs to match the anticipated morphology of the donor tooth (Figure 1E).

Sticky bone was prepared according to the protocol described above and placed in the distal and buccal osseous defects of the recipient site (Figure 1F). The sticky bone was packed into the defect adjacent to the mesial root of tooth 47 and the buccal bone defect of the 46 region to augment the compromised recipient site (Figure 1G). Tooth 48 was then atraumatically and completely extracted, with careful preservation of the periodontal ligament (Figure 1H). Calculus and soft deposits on the cervical region of the donor tooth were removed. The donor tooth was inserted into the prepared recipient socket at the tooth 46 site and stabilized with combined suture and ligature-wire fixation (Figure 1I).

#### Postoperative management and follow-up

Immediate postoperative CBCT confirmed appropriate positioning of tooth 48 at the tooth 46 recipient site and showed an approximately 2-mm-thick layer of radiopaque graft material at the distal and buccal aspects of the transplanted tooth (Figures 1P,Q). Endodontic access was performed 2 weeks postoperatively, and definitive root canal treatment was completed 3 months after transplantation. Periapical radiography confirmed adequate root canal obturation (Figure 1R).

At the 1-year follow-up, the transplanted tooth remained asymptomatic. The periodontal soft tissues were healthy, the periodontal soft tissues appeared clinically healthy, no percussion tenderness or abnormal metallic percussion sound was detected, and no abnormal mobility was observed (Figures 1K,L). Radiographic examination demonstrated periradicular bone healing and maintenance of a visible periodontal ligament space. Root-surface changes were observed; however, no clear progression of inflammatory or replacement resorption, ankylosis-related changes, or progressive periapical pathology was identified during the 12-month follow-up (Figure 1S).

### Case 2

#### Patient information and chief complaint

A 23-year-old woman presented with a long-standing history of a severely compromised left mandibular posterior tooth. She requested consultation regarding the feasibility of autogenous tooth transplantation.

#### Medical and dental history, clinical and radiographic examination

Her medical history was unremarkable, with no history of hypertension, diabetes mellitus, or drug allergy. Extraoral examination showed symmetrical facial contours, normal mouth opening and mandibular movement, and no temporomandibular joint tenderness or clicking.

**FIGURE 2:**
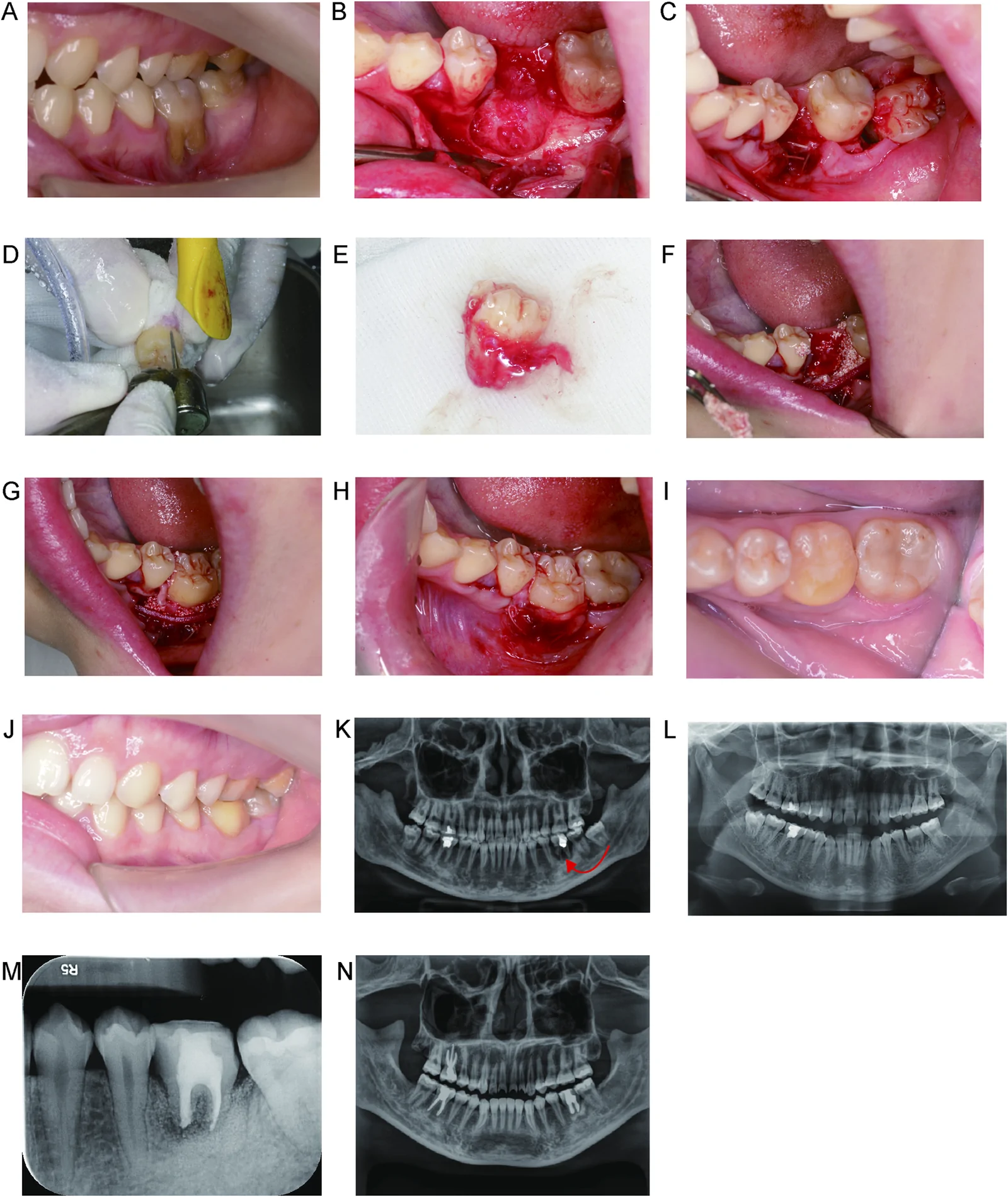
Clinical photographs and radiographic images of Case 2 showing autogenous tooth transplantation of tooth 38 to the tooth 36 recipient site. **(A)** Initial intraoral photograph of tooth 36. **(B)** Minimally traumatic extraction of tooth 36. **(C)** Minimally traumatic extraction of donor tooth 38. **(D)** Removal of calculus from the cervical region of tooth 38. **(E)** Photograph of the extracted donor tooth 38. **(F)** Placement of sticky bone into the prepared recipient socket at the tooth 36 site after socket refinement. **(G)** Insertion of tooth 38 into the prepared tooth 36 recipient socket. **(H)** Stabilization of transplanted tooth 38 at the tooth 36 site. **(I,J)** Intraoral photographs at the 1-year follow-up after autogenous tooth transplantation. **(K)** Preoperative CBCT image before autogenous tooth transplantation. **(L)** Immediate postoperative panoramic radiograph after transplantation. **(M)** Periapical radiograph after completion of root canal treatment 3 months after transplantation. **(N)** CBCT image at the 1-year follow-up. ATT, autogenous tooth transplantation; CBCT, cone-beam computed tomography.

Intraoral examination revealed a large disto-occlusal restoration on tooth 36. The tooth was tender to percussion and exhibited grade II mobility. Circumferential periodontal probing depths of 9–10 mm were recorded around tooth 36 (Figure 2A). Tooth 38 was present and showed no obvious clinical abnormality. CBCT demonstrated severe alveolar bone loss around tooth 36, extending to the apical region (Figure 2K).

#### Diagnosis

Tooth 36: residual root with chronic apical periodontitis.

Tooth 38: nonfunctional third molar.

#### Treatment options and decision-making

Three treatment alternatives were discussed: extraction of tooth 36 followed by orthodontic mesialization of tooth 38 to close the space; extraction of tooth 36 with simultaneous alveolar ridge preservation followed by delayed implant-supported restoration after 3 months; or extraction of tooth 36 and autogenous transplantation of tooth 38 into the 36 site.

Because orthodontic space closure would require a prolonged treatment period and implant therapy would impose a greater financial burden, the patient selected autogenous transplantation. The treatment plan included extraction of tooth 36, reconstruction of the recipient-site defect with sticky bone, simultaneous transplantation of tooth 38, endodontic access 2 weeks after transplantation, definitive root canal treatment after stabilization of tooth mobility, and serial follow-up at 2 weeks, 4 weeks, 3 months, and 1 year.

#### Surgical procedure

A full-thickness mucoperiosteal flap was elevated, revealing a circumferential vertical osseous defect around the 36 socket (Figure 2B). The recipient bed was prepared with surgical burs to accommodate the donor tooth. Venous blood was collected and centrifuged to prepare sticky bone, which was then packed into the osseous defect to augment the compromised ridge (Figure 2F).

Tooth 38 was atraumatically extracted with preservation of the root surface and periodontal ligament (Figures 2C,E). The cervical region of the donor tooth was carefully debrided of calculus and soft deposits (Figure 2D). The tooth was then transplanted into the prepared and augmented 36 recipient socket (Figure 2G). Because the prepared socket closely matched the donor root morphology and adequate primary stability was obtained, suture fixation alone was used, without rigid fixation (Figure 2H).

#### Postoperative management and follow-up

Immediate postoperative imaging demonstrated satisfactory graft adaptation around the transplanted tooth and appropriate positioning of tooth 38 in the 36 region (Figure 2L). Endodontic access was performed 2 weeks postoperatively, and definitive root canal treatment was completed at 3 months. Periapical radiography showed dense and adequate root canal obturation (Figure 2M).

At the 1-year follow-up, the transplanted tooth was clinically stable and asymptomatic. The surrounding periodontal soft tissues were healthy, no abnormal mobility was present, and the surrounding periodontal soft tissues appeared clinically healthy (Figures 2I,J). Radiographic examination showed stable surrounding alveolar bone and a visible periodontal ligament space. Root-surface changes were observed; however, no clear progression of inflammatory or replacement resorption, ankylosis-related changes, or progressive periapical pathology was identified during the 12-month follow-up (Figure 2N).

### Case 3

#### Patient information and chief complaint

A 23-year-old woman presented with an unrestorable right maxillary posterior root remnant associated with intermittent discomfort in the ipsilateral nasal cavity for several years. She requested extraction of the residual root and consultation regarding autogenous tooth transplantation.

#### Medical and dental history, clinical and radiographic examination

The patient’s medical history was noncontributory. She denied hypertension, diabetes mellitus, and drug allergy. Extraoral examination revealed symmetrical facial contours, normal mouth opening and mandibular movement, and no temporomandibular joint tenderness or clicking.

**FIGURE 3:**
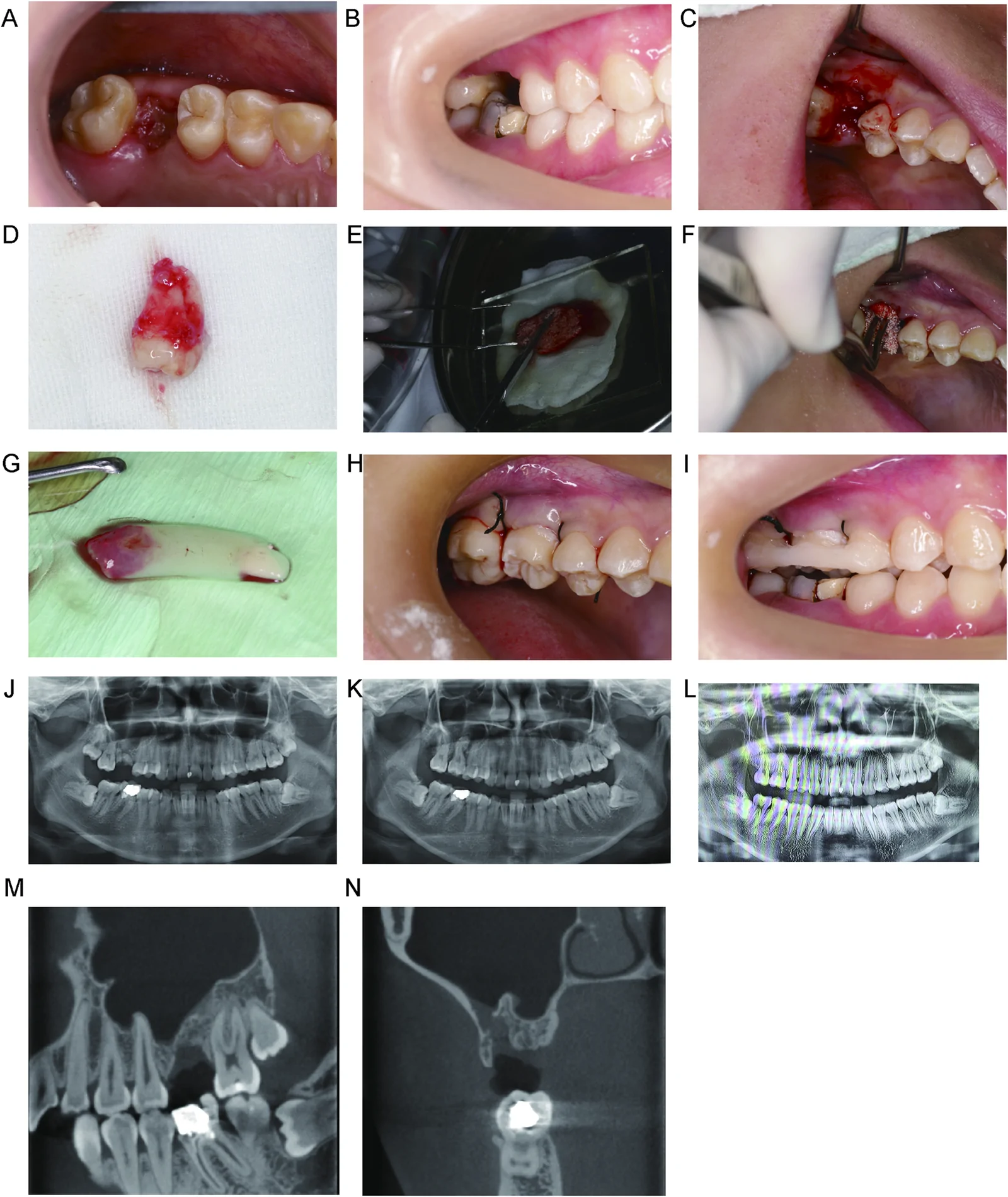
Clinical photographs and radiographic images of Case 3 showing autogenous tooth transplantation of tooth 18 to the tooth 16 recipient site with sinus-floor defect management. **(A,B)** Initial intraoral photographs of tooth 16. **(C)** Minimally traumatic extraction of tooth 16. **(D)** Minimally traumatic extraction of donor tooth 18. **(E)** Preparation of sticky bone. **(F)** Placement of sticky bone into the alveolar socket defect at the tooth 16 recipient site. **(G)** Preparation of concentrated growth factor. **(H and I)** Stabilization of transplanted tooth 18 at the tooth 16 recipient site. **(J)** Preoperative panoramic radiograph before autogenous tooth transplantation. **(K)** Immediate postoperative panoramic radiograph after transplantation. **(L)** Panoramic radiograph at the 1-year follow-up. **(M,N)** Preoperative CBCT before autogenous tooth transplantation. ATT, autogenous tooth transplantation; CGF, concentrated growth factor.

Intraoral examination showed that tooth 16 was reduced to a subgingival residual root (Figures 3A,B). The surrounding gingiva was erythematous and swollen, and mild swelling was present in the buccal vestibule. CBCT showed that the root remnant of tooth 16 was closely related to the maxillary sinus, with loss of the intervening bony sinus floor and vertical loss of the palatal bony wall (Figures 3M,N).

#### Diagnosis

Tooth 16: residual root with chronic apical periodontitis.

Tooth 18: nonfunctional third molar.

Suspected odontogenic maxillary sinus involvement associated with tooth 16.

#### Treatment options and decision-making

Three treatment options were proposed: extraction of tooth 16 followed by orthodontic mesialization of tooth 18 to close the space; extraction of tooth 16 with simultaneous alveolar ridge preservation followed by delayed implant-supported restoration after 3 months; or extraction of tooth 16 followed by autogenous transplantation of tooth 18 into the 16 site.

The patient chose autogenous transplantation because of the prolonged treatment duration associated with orthodontic therapy and the higher cost of implant treatment. However, unlike the mandibular posterior cases, this case involved loss of the bony sinus floor adjacent to the tooth 16 recipient site, with anticipated exposure of the Schneiderian membrane. Therefore, a staged protocol was selected. The plan consisted of atraumatic extraction of tooth 16, a short healing period to allow resolution of the local inflammatory condition and improvement of the sinus-related symptoms, limited sinus-floor elevation and recipient-site augmentation with sticky bone, simultaneous transplantation of tooth 18 into the tooth 16 recipient site, postoperative endodontic management, and regular follow-up. No otorhinolaryngological consultation was obtained; the assessment was based on clinical symptoms and dental CBCT findings.

#### Surgical procedure

In the first stage, tooth 16 was atraumatically extracted (Figure 3C). The socket was closed with nonresorbable sutures, and hemostasis was achieved with cotton-roll compression for 30 min.

Two weeks later, a full-thickness mucoperiosteal flap was elevated. Loss of the bony sinus floor was identified at the tooth 16 recipient site, resulting in exposure of the Schneiderian membrane. The membrane remained intact, and no mucosal perforation or oroantral communication was identified. The recipient socket was carefully prepared while avoiding injury to the exposed Schneiderian membrane. The intact membrane was gently elevated, and sticky bone prepared according to the protocol described above was placed beneath the elevated membrane and around the deficient recipient site to support limited sinus-floor elevation and recipient-site augmentation (Figures 3E,F).

Tooth 18 was then atraumatically and completely extracted (Figure 3D). After removal of cervical deposits, the donor tooth was transplanted into the prepared 16 recipient socket. Because of the reduced bony support at the maxillary recipient site, the transplanted tooth was stabilized with metal wire fixation (Figures 3H,I).

#### Postoperative management and follow-up

Immediate postoperative imaging demonstrated the augmented sinus-floor contour and appropriate positioning of tooth 18 in the tooth 16 recipient site (Figure 3K). Definitive root canal treatment was completed 3 months after transplantation, and radiographic examination confirmed adequate obturation.

At the 1-year follow-up, the transplanted tooth remained asymptomatic and clinically functional. The patient reported no recurrence of nasal or sinus-related discomfort. The periodontal soft tissues were healthy, no deep periodontal pocket was detected, no percussion tenderness or abnormal metallic percussion sound was detected, and no abnormal mobility was observed. Comparison of the immediate postoperative and 12-month radiographs demonstrated maintenance of the augmented sinus-floor contour without obvious displacement or loss of the grafted region. A visible periodontal ligament space was present around the transplanted tooth. Root-surface changes were observed; however, no clear progression of inflammatory or replacement resorption, ankylosis-related changes, or progressive periapical pathology was identified (Figure 3L).

### Case 4

#### Patient information and chief complaint

An 18-year-old woman presented with failed surgical-orthodontic eruption of an impacted left maxillary central incisor. Approximately 18 months earlier, she had undergone labial surgical exposure and orthodontic traction of tooth 21 for intraosseous impaction. Despite repeated orthodontic activation, the eruption of tooth 21 remained minimal. She was therefore referred to our department for evaluation of autogenous tooth transplantation.

#### Medical and dental history, clinical and radiographic examination

**FIGURE 4:**
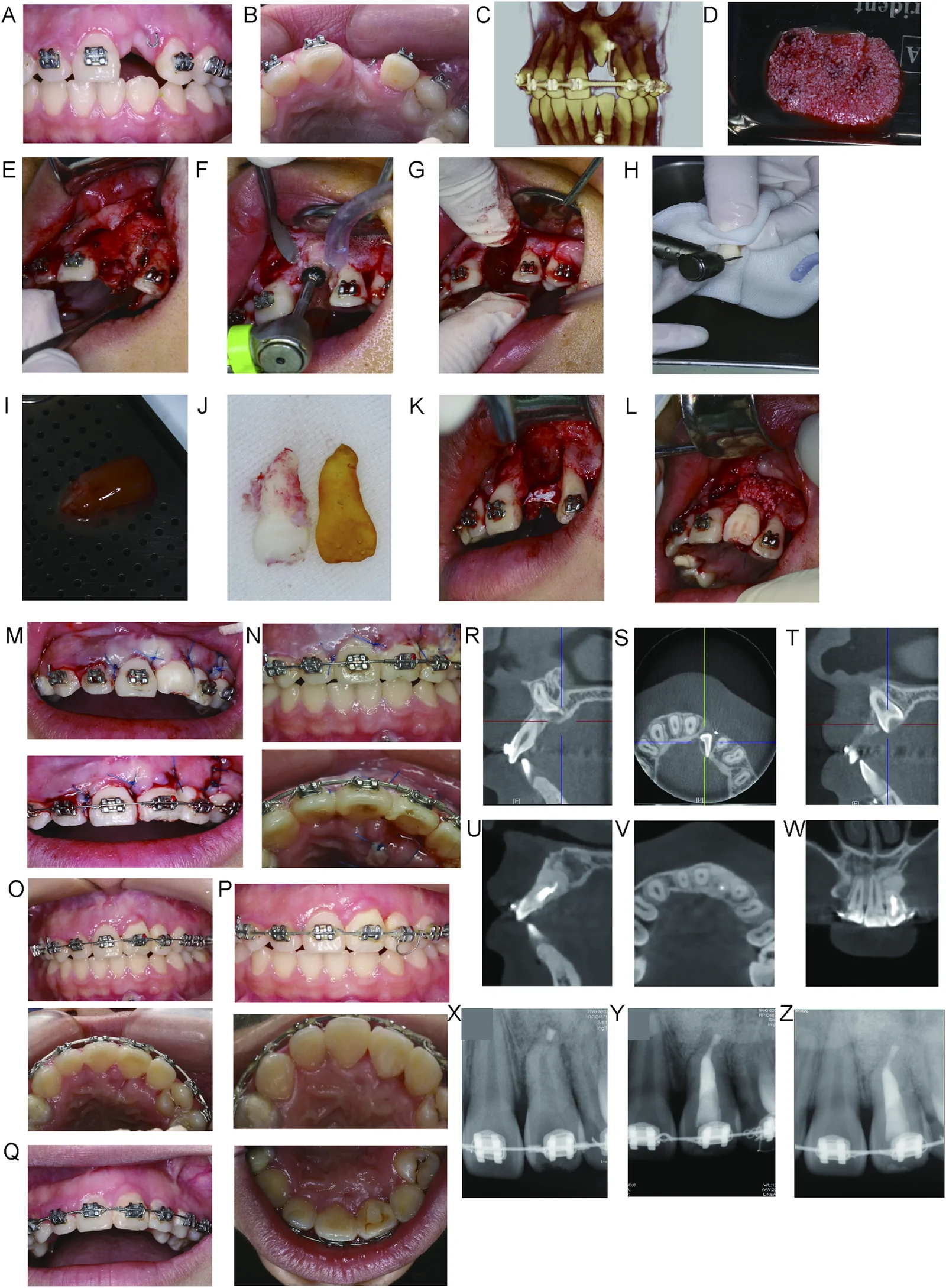
Clinical photographs and radiographic images of Case 4 showing digitally assisted autogenous transplantation of impacted tooth 21 to the maxillary anterior region. **(A–C)** Initial intraoral photographs. **(D)** Preparation of sticky bone. **(E)** Elevation of the labial mucoperiosteal flap and exposure of the recipient site. **(F, G)** Atraumatic extraction of impacted tooth 21. **(H)** Deliberate reduction of the interfering root prominence, root-end preparation, and retrograde filling. **(I)** Preparation of concentrated growth factor. **(J)** Comparison of the 3D-printed donor-tooth replica with the extracted tooth. **(K)** Placement of the resorbable collagen membrane. **(L)** Placement of sticky bone and transplantation of tooth 21 into the prepared recipient site. **(M)** Immediate postoperative intraoral photograph. **(N)** Intraoral photograph at 4 days. **(O)** Intraoral photograph at 7 days. **(P)** Intraoral photograph at 5 months. **(Q)** Intraoral photograph at 30 months. **(R–T)** Preoperative CBCT images. **(U–W)** CBCT images at the 30-month follow-up. **(X)** Immediate postoperative periapical radiograph. **(Y)** Periapical radiograph at the 5-month follow-up. **(Z)** Periapical radiograph at the 17-month follow-up. ATT, autogenous tooth transplantation; CBCT, cone-beam computed tomography; CGF, concentrated growth factor; 3D, three-dimensional.

Her medical history was notable for glucose-6-phosphate dehydrogenase deficiency. She denied hypertension and drug allergy. Intraoral examination showed that tooth 21 remained unerupted, with an orthodontic traction appliance visible (Figures 4A–C). CBCT demonstrated deep intraosseous impaction of tooth 21 with an associated bony defect. The tooth exhibited marked root dilaceration and palatal displacement of the crown (Figures 4R,S).

#### Diagnosis

Tooth 21: Impacted tooth 21 (failed surgical-orthodontic traction).

#### Treatment options and decision-making

Because previous orthodontic traction had been unsuccessful, two treatment options were proposed: extraction of tooth 21 followed by staged guided bone regeneration and implant-supported restoration after skeletal and periodontal conditions became more favorable; or extraction of tooth 21 with immediate autogenous transplantation into the anatomically correct maxillary central incisor position.

Considering the patient’s young age and the anticipated esthetic difficulty of future implant rehabilitation in the anterior maxilla if labial bone resorption progressed, the patient and her family selected the transplantation approach. This strategy offered the possibility of preserving a natural tooth in the esthetic zone while avoiding, or at least delaying, implant therapy.

#### Surgical procedure

A digitally assisted surgical protocol was used. The CBCT dataset was exported in Digital Imaging and Communications in Medicine (DICOM) format and imported into Mimics software for segmentation and three-dimensional reconstruction of tooth 21. After completion of the segmentation and verification of the reconstructed model, the donor-tooth model was exported in stereolithography (STL) format for fabrication of a three-dimensional printed replica. The replica was subsequently used to assist recipient-site preparation before extraction of the actual donor tooth (Figure 4J).

After venous blood collection and preparation of sticky bone, a full-thickness mucoperiosteal flap was elevated through a labial approach to expose the impacted tooth and the deficient recipient alveolar bed in the ideal position of the maxillary left central incisor (Figure 4E). The recipient socket was prepared with surgical burs, and the 3D-printed replica was repeatedly tried in to refine the socket morphology and achieve a precise, minimally traumatic fit (Figure 4J). Once the recipient site had been optimized, sticky bone was placed into the prepared socket.

Tooth 21 was then atraumatically extracted through the labial approach (Figures 4F,G). During the extraoral procedures, the donor tooth was kept hydrated with sterile saline. No unintended root-surface damage was observed during extraction. Because of the marked root dilaceration, the interfering root prominence was subsequently reduced in a limited and deliberate manner to facilitate insertion into the prepared recipient socket. Root-end preparation was performed using an ultrasnic retrograde instrument to a depth of approximately 3 mm, followed by retrograde filling with iRoot BP Plus. Retrograde filling was performed to obtain an apical seal because the severe root dilaceration would have made subsequent apical management difficult after transplantation (Figure 4H). Sticky bone was placed within the alveolar defect surrounding the prepared recipient socket and covered with a Bio-Gide® resorbable collagen membrane (Geistlich Pharma AG, Switzerland). The prepared tooth was then transplanted into the recipient socket in the planned anterior position and stabilized using a conventional stainless-steel orthodontic ligature wire. The fixation was removed 7 days after transplantation after satisfactory clinical stability had been confirmed (Figure 4L). The total extraoral time, measured from complete extraction of tooth 21 to its insertion into the prepared recipient socket, did not exceed 8 min.

#### Postoperative management and follow-up

Immediate postoperative periapical radiography confirmed that tooth 21 had been repositioned adjacent to tooth 11 in the planned position and showed satisfactory placement of the root-end filling (Figure 4X). At 4 and 7 days postoperatively, the gingival tissues at the surgical site showed uneventful healing (Figures 4N,O).

Orthograde root canal treatment was initiated 7 days after transplantation, and Vitapex was placed as an intracanal medicament. Because the patient was unable to attend the scheduled appointments for personal reasons, definitive root canal obturation was completed 5 months after transplantation (Figure 4Y). Thus, root canal treatment was initiated during the early postoperative period, whereas definitive obturation was delayed. At the 17-month follow-up, periapical radiography showed retention of the transplanted tooth and persistent root-surface changes (Figure 4Z). At the 30-month follow-up, the tooth remained asymptomatic and clinically functional, with physiological mobility, healthy periodontal soft tissues, and no abnormal metallic percussion sound (Figure 4Q). Comparison of the available serial periapical radiographs and the 30-month CBCT images showed no definite progression of inflammatory or replacement resorption, ankylosis-related changes, or periapical pathology; however, subtle progression could not be excluded because the serial imaging protocol was not standardized (Figures 4U–W).

## Discussion

The biological outcome of ATT depends principally on the healing potential of the PDL. Viable PDL cells on the donor root surface are essential for periodontal reattachment, cementum repair, and re-establishment of a functional PDL space after transplantation (Chugh et al., 2012). Atraumatic extraction, preservation of the root surface, and minimization of extraoral time are therefore fundamental, because prolonged exposure and repeated manipulation may reduce PDL cell viability and compromise periodontal healing (Lee and Kim, 2012). Root development also influences healing: pulpal revascularization is more predictable in teeth with incomplete root formation, whereas mature teeth with closed apices are more susceptible to pulp necrosis and generally require planned endodontic treatment (Rohof et al., 2018).

Recipient-site anatomy represents another important determinant of healing. Vertical bone loss, buccal plate deficiency, irregular socket morphology, or sinus-floor bony defects may reduce socket adaptation and mechanical support. Adequate primary stability and appropriate adaptation between the donor root and recipient socket are important because excessive mobility, inadequate alveolar support, and uncontained defects are associated with unfavorable healing and transplant failure (Yang, Jung, and Pang, 2019; Zhao et al., 2021). In the present cases, sticky bone was used to manage peri-root defects rather than to replace PDL-mediated healing. Its cohesive and moldable fibrin-bound structure may facilitate placement and stabilization of particulate graft material within irregular defects (Rupawala et al., 2020; Zhao et al., 2021). However, these are primarily material-handling properties, and direct evidence that sticky bone improves ATT outcomes remains limited.

Endodontic management of mature donor teeth should be planned while minimizing additional trauma to the PDL. Because spontaneous pulpal revascularization is uncommon in teeth with complete root formation, root canal treatment is generally recommended to reduce the risk of pulp necrosis and inflammatory root resorption (Chhana et al., 2024; Liao et al., 2023). Reported approaches include preoperative treatment, extraoral treatment during transplantation, and delayed postoperative treatment, generally within the first several weeks depending on clinical stability (Azevedo et al., 2007; Boschini et al., 2020). In the present series, postoperative endodontic treatment was selected to avoid prolonging extraoral manipulation. Digitally planned or replica-assisted socket preparation may further reduce repeated insertion of the actual donor tooth and thereby limit extraoral handling (Bae et al., 2010; Lee and Kim, 2012).

ATT outcomes should be evaluated using both clinical and radiographic parameters rather than radiographic bone fill alone. Periodontal healing requires healthy gingival tissues, physiological mobility, acceptable probing findings, and the absence of progressive root resorption or ankylosis (Kamata et al., 2021; Rohof et al., 2018). Keratinized tissue width may also contribute to long-term peri-dental and peri-implant soft-tissue stability, and autogenous soft-tissue grafting remains a predictable option when augmentation is indicated (Bertl et al., 2017; Rotundo et al., 2024). Tooth-gingival transplantation has also been described for selected ATT situations requiring combined tooth and soft-tissue management (Suzuki et al., 2018). In the present cases, the peri-transplant soft tissues appeared clinically healthy during follow-up; however, keratinized and attached gingival widths were not measured using a standardized protocol. Therefore, no conclusion can be made regarding soft-tissue augmentation associated with ATT or sticky bone.

Case 3 involved a compromised maxillary posterior recipient site with loss of the bony sinus floor associated with chronic odontogenic inflammation. During surgery, the Schneiderian membrane was exposed but remained intact, and no oroantral communication or mucosal perforation was identified. A limited sinus-floor elevation was therefore performed, and sticky bone was placed beneath the elevated membrane to augment the recipient site and provide additional osseous support for the transplanted tooth. Postoperative imaging showed maintenance of the reconstructed sinus-floor contour, and no sinus-related symptoms were reported during follow-up. However, because this was a single case and no comparison procedure was available, the contribution of sticky bone to sinus-floor augmentation or transplantation healing cannot be determined.

This study has several limitations, including the small sample size, retrospective and purposive case selection, absence of a control group, heterogeneous clinical conditions, and follow-up periods of 12–30 months. Clinical and radiographic assessments were not fully standardized, which limited the quantitative comparison of root-surface changes and the detection of subtle progression, and the contribution of sticky bone cannot be separated from other determinants of ATT healing, including PDL preservation, recipient-site preparation, primary stability, infection control, endodontic management, and postoperative care. The present cases therefore illustrate the technical use of sticky bone in compromised recipient sites but do not establish its effectiveness or superiority. Controlled studies with standardized protocols, objective outcome assessment, and longer follow-up are required.

## Conclusion

Autogenous tooth transplantation with sticky bone–assisted recipient-site management was feasible in the four presented cases, and all transplanted teeth remained clinically functional during follow-up periods of 12–30 months. Radiographic root-surface changes were observed, but no clear progression of inflammatory or replacement resorption was identified during the available observation periods. However, the contribution of sticky bone cannot be determined because of the small sample size, case heterogeneity, non-standardized radiographic assessment, and absence of a control group.

## Data Availability

The original contributions presented in the study are included in the article/supplementary material, further inquiries can be directed to the corresponding author.
